# How do maternal emotion and sleep conditions affect infant sleep: a prospective cohort study

**DOI:** 10.1186/s12884-022-04504-6

**Published:** 2022-03-23

**Authors:** Xuemei Lin, Ronghui Zhai, Jiafeng Mo, Jingzhou Sun, Peishan Chen, Yuejun Huang

**Affiliations:** 1 Department of Neonatology, Second Affiliated Hospital of Shantou University Medical College, North Dongxia Road, Shantou, 515041 Guangdong China; 2 Department of Neonatology, Shenshan Central Hospital of Sun Yat-sen Memorial Hospital, Sun Yat-sen University, Shanwei, 516600 Guangdong China; 3 Department of Mathematics, Shantou University Science College, College Road, Shantou, 515041 Guangdong China; 4 Department of Obstetrics, Second Affiliated Hospital of Shantou University Medical College, North Dongxia Road, Shantou, 515041 Guangdong China

**Keywords:** Mother, Infant, Emotion, Sleep disorder, Glucocorticoid receptor, Melatonin receptor

## Abstract

**Background:**

Recent studies suggest that the incidence of infant sleep disorder is related to maternal emotional and sleep conditions, but how they influence each other is not fully understood.

**Methods:**

A total of 513 pairs of parents and infants were enrolled in this prospective cohort study. Maternal emotional and sleep conditions were assessed using a self-rating depression scale, self-rating anxiety scale, and Pittsburgh Sleep Quality Index at the third trimester and within 3 months after delivery. Infant sleep was assessed by the Brief Screening Questionnaire for Infant Sleep Problems within 3 months after birth. Expression of the glucocorticoid receptor (GR), melatonin receptors (MR), exchange proteins directly activated by cAMP (EPAC) receptors, and dopamine receptor (DR) in the placenta was detected by immunohistochemistry. Methylation of the promoter regions for the GR (NR3C1 and NR3C2), MR (MTNR1A and MTNR1B), EPAC (RASGRF1 and RASGRF2), and DR (DRD1 and DRD2) genes was assessed by next generation sequencing-based bisulfite sequencing PCR.

**Results:**

The incidence of sleep disorders in infants 0–3 months of age in this cohort was 40.5%. Risk factors for infant sleep disorder were low education level of the father, depression of father, maternal postpartum depression, postpartum anxiety, postpartum sleep disorder, and maternal sleep disorder extend from the third trimester to postpartum. There was no difference in expression of placental DR, GR, MR, and EPAC between mothers whose infants were with and without sleep disorders. Methylation of MTNR1B was higher and expression of MR was lower in the placenta of mothers with sleep disorder in the third trimester than in mothers without sleep disorder. Level of NR3C2 methylation was lower and GR expression was higher in the placenta of mothers with sleep disorder extend from the third trimester to postpartum than in mothers without sleep disorder.

**Conclusion:**

Maternal sleep disorders in the third trimester could lead to decreased MR expression by up-regulating MTNR1B methylation, and then resulting in elevated cortisol and increased GR expression by down-regulating NR3C2 methylation, which could increase the incidence of maternal postpartum sleep disorders, finally, the maternal postpartum sleep disorder could result in the high incidence of infant sleep disorder.

## Background

Good sleep is important for the physical and neurobehavioral development of infants. Infants whose mothers had depression slept less, had a longer sleep latency period, and were more likely to wake up two or more times during the night [1]. Sleep disruption in infants can lead to sleep disruption in mothers [2], which can increase the risk of postpartum depression [3]. Studies have shown that maternal emotional or sleep disorders during pregnancy and postpartum are closely associated with sleep disorders in infants [4, 5], but how they influence each other is not fully understood.

Several studies show that the cortisol level is higher in pregnancy or postpartum women with emotional or sleep disorders [6]. Melatonin acts on melatonin receptor (MR) to promote sleep and prevent depression. Studies have found that elevated blood cortisol levels can be decreased after treatment of chronically stressed mice with melatonin [7], and melatonin can reduce the affinity of glucocorticoid receptor (GR) [8]. Moreover, melatonin and MRs may inhibit cortisol production through exchange proteins directly activated by cAMP (EPAC) receptor [9]. Dopamine is one of the neurotransmitters secreted by the placenta, is involved in placenta circulation and affects fetal brain development [10]. Dopamine and dopamine receptor (DR) are also involved in the occurrence of depression and sleep. Placenta involved in the synthesis of hormones during pregnancy [11]. Placental epigenetic, which are susceptible to maternal environment, including physical and psychological disorders [12], can functionally regulate gene expression [13]. Therefore, we speculated that the expression of GR, MR, EPAC and DR in the placenta may be involved in the development of maternal emotion and sleep disorders during pregnancy and postpartum, leading to increased risk of infant sleep disorders.

Fathers and mothers play an equally important role in the development of their children. The incidence of postpartum depression in fathers is about 8.4–28%. However, there are few studies that include the father’s emotional and sleep status into the analysis of the risk factors of infant sleep. And few comprehensive studies have addressed the effects of maternal emotion or sleep disorders which extend from the third trimester of pregnancy to postpartum on infant sleep. Therefore, there are three aims in this study. First, we use a prospective cohort study to analyze the effects of maternal emotion and sleep disorder which extend from the third trimester of pregnancy to postpartum on infant’ sleep. Second, we use a cross-sectional study to analyze the effects of the father’s emotional and sleep status on infant’ sleep. Third, we use a nested case-control study to detect GR, MR, EPAC, and DR protein expression and methylation of the promoter regions for GR (NR3C1 and NR3C2), MR (MTNR1A and MTNR1B), EPAC (RASGRF1 and RASGRF2), and DR (DRD1 and DRD2) genes in the placenta, to further analyze the relationship between maternal emotional or sleep conditions and infant sleep disorder.

## Methods

### Ethics statement

This study was conducted in compliance with the Declaration of Helsinki, and with the approval of the ethics committees of the Second Affiliated Hospital of Shantou University Medical College. Informed consent was obtained from all participating mothers.

### Setting of the study

This is a prospective cohort study. Data collection was performed in the Women and Children Health Care Center in the Second Affiliated Hospital of Shantou University Medical College from April 2019 to December 2020. The time of participant’s enrollment is at the first trimester of pregnancy. The inclusion criteria of pregnant women were as follows: (1) singleton pregnancy, (2) intention to have regular antenatal care and give birth in our hospital, and (3) able to understand the relevant scale options in this subject. Pregnant women and their infants were excluded from this study if they met the following criteria: (1) did not give birth in our hospital, (2) had a preterm birth, (3) had thyroid, liver, kidney, lung or heart disease before and after pregnancy, (4) either parent of the infant had depression, anxiety disorder, somnipathy, schizophrenia, mania, dissociative personality disorder, or other mental disorders prior to pregnancy, (5) infants had a congenital malformation, (6) data was incomplete.

### Data collection

At the first visit for antenatal care, pregnant women were required to complete a form, which was used to collect variables, including their age, height, body weight before pregnancy, education level, family income, history of adverse pregnancy, passive smoking, residential area, and the father’s age, height, body weight, and education level. After delivery, we collected the data from the medical record system, including anemia during pregnancy, hypertensive disorder complicating pregnancy (HDCP), pregnancy diabetes, method of delivery, neonatal asphyxia, gestational age (GA), birth weight of the infants, and gender of infants. At the first visit after delivery for child health care of infants, mothers were required to complete a questionnaire, which was used to collect variables of the neonate, including hospitalization of the neonate, allergic disease, jaundice, phototherapy, duration of exposure to sunlight each day, and vitamin D intake.

### Assessment of emotion and sleep conditions

Pregnant women filled out a self-rating depression scale (SDS), the Self-Rating Anxiety Scale (SAS), and Pittsburgh Sleep Quality Index (PSQI) at both the third trimester and within 3 months after delivery. The baby’s father also filled out the SDS, SAS, and PSQI within 3 months after baby birth. Infant sleep was assessed by the Brief Screening Questionnaire for Infant Sleep Problems (BISQ) [14] and was completed by the mother for the first child health care within 3 months after birth. The SDS, SAS, and PSQI of parents, and BISQ of infants, collected within 3 months after baby delivery, were filled out at the same time.

The SDS consists of 20 items, including two items of psycho-emotional symptoms, eight items of physical disorders, two items of psychomotor disorders, and eight items of depressive psychological disorders [15]. The score for each item ranges from 1 to 4. Then the total score is multiplied by 1.25 to obtain a standard SDS score. A score ≥ 53 indicates depression. The total reliability coefficient of the SDS is 0.784 (Cronbach’s alpha) in Chinese women living in a rural area, and it has been shown to be a valid and efficient tool for screening depression in Chinese population [15].

The SAS is a 20-item self-administered scale to measure anxiety [16]. Each question is scored on a scale of 1 to 4 (rarely, sometimes, frequently, and always). The total score ranges between 20 and 80, which is multiplied by 1.25 to obtain a standard SAS score. A score ≥ 50 indicates anxiety. The SAS is a valid tool with good internal consistency (Cronbach’s alpha, 0.897) and widely used to screen anxiety.

Sleep quality of the parents was assessed using the PSQI [17], which consists of 19 self-rated questions and 5 questions rated by the bed partner. The 19 items are grouped into scores with the seven following components: subjective sleep quality, sleep latency, sleep duration, sleep efficiency, sleep disturbances, use of sleep medication, and daytime dysfunction. These component scores are added to a global PSQI score with a range of 0 to 21, with higher scores indicating worse sleep quality. A PSQI score above 5 is abnormal.

The questionnaire variables in the BISQ included 1) nocturnal sleep duration (between the hours of 7 PM and 7 AM); 2) daytime sleep duration (between the hours of 7 AM and 7 PM); 3) number of times of waking during the night; 4) duration of wakefulness during the night hours (10 PM to 6 AM); 5) nocturnal sleep-onset time (the clock time at which the child falls asleep for the night); 6) settling time (latency to falling asleep for the night); 7) method of falling asleep; 8) location of sleep; 9) preferred body position; 10) age of child; 11) gender of child; 12) birth order; and 13) role of responder (who completed the BISQ).

### Diagnosis of infant sleep disorder

According to “A Brief Screening Questionnaire for Infant Sleep Problems” published in Pediatrics [14], waking three times or having a duration of wakefulness greater than 1 h, or total sleep time less than 9 h for each 24 h is indicative of infant sleep disorder.

### Placenta sample collection

Approximately 10 g tissue from the maternal side of the placenta in each participant was obtained immediately after delivery. Samples of placental parenchyma were carefully dissected by trained research assistants to assure the maternal decidua was separated from the sample to enable extraction of DNA of fetal origin. The samples were divided into two parts. One part was frozen in liquid nitrogen and stored at − 80 °C for methylation detection. The remainder was placed in 4% paraformaldehyde in 0.1 M phosphate buffer (pH 7.4) for immunohistochemistry.

### Immunohistochemistry

Tissues were processed by dehydration, clearing and paraffin imbedding, then cut into 5 μm thick sections. Five sections were taken from each placenta for immunostaining with mouse anti-human GR, MR, EPAC, and DR2 monoclonal antibodies (Sigma, USA). Antibodies were diluted 1:200 in 3% BSA and incubated with tissue section overnight at room temperature. Detection was carried out using a 1:200 dilution in 3% BSA of biotin-labeled sheep anti-mouse IgG (Sigma, USA), then SA-HRP (Sigma, USA) was added for 1 h at room temperature. Finally, the sections were stained with DAB/H_2_O_2_ (Sigma, USA). Cells positively stained for GR, MR, EPAC, and DR2 in the villous cytotrophoblast (vCTB), syncytiotrophoblast (STB), and extravillous trophoblast (EVT) of placenta were measured and analyzed using an Image-Pro Plus Version 5.0 color image analysis system. The numbers of immunoreactive cells were counted in five high power fields in each section of the villous cytotrophoblast (vCTB), syncytiotrophoblast (STB), and extravillous trophoblast (EVT) regions of the placenta.

### Methylation detection

Methylation levels of the GR (NR3C1 and NR3C2), MR (MTNR1A and MTNR1B), EPAC (RASGRF1 and RASGRF2), and DR (DRD1 and DRD2) gene promoters were assessed by next generation sequencing-based bisulfite sequencing PCR (NGS-BSP) [18]. BSP primers were designed using the online Meth-Primer website. DNA samples were extracted using a QI Amp DNA Mini Kit (Qiagen, Inc.). Purified DNA was quantified using an ND-1000 spectrophotometer (Nanodrop), and DNA samples (1 μg) were bisulfite-modified using an EZ DNA Methylation Kit (Zymo Research). For each sample, BSP products of the target genes were generated, pooled equally, and subjected to adaptor ligation. Barcoded libraries from all samples were sequenced on the Illumina HiSeq platform using a paired-end 150 bp strategy. Data for the genes is listed in Table 1.

**Table 1:** Data of the genes detected for methylation

Gene	Position	Size	Genetic belt
NR3C1	chr5:143,277,931-143,435,512	157,582	5q31.3
NR3C2	chr4:148,078,762-148,444,698	365,937	4q31.23
MTNR1A	chr4:186,532,769-186,555,567	22,799	4q35.2
MTNR1B	chr11:92,969,651-92,986,241	13,132	11q14.3
DRD1	chr5:175,440,036-175,444,182	4147	5q35.2
DRD2	chr11:113,409,605-113,475,691	66,087	11q23.2
RASGRF1	chr15:78,959,906-79,090,780	130,875	15q25.1
RASGRF2	chr11:64,726,911-64,745,481	18,571	11q13.1

### Statistical analysis

For continuous variables, the Shapiro-Wilk test was used to determine the normal distribution of the continuous variables, and the Wilcoxon-Mann-Whitney U-test was conducted for skewed distributions (presented as the median and the interquartile range*).* Descriptive statistics for categorical variables were reported as frequency (percentage) and compared using the Pearson chi-square test or Fisher’s exact test, as appropriate. The variables with *p*-values less than 0.1 were analyzed by logistic regression. Before logistic analysis, the correlation of all the variables were assessed by Spearman correlation analysis. For logistic regression used to examine the risk factors of infant sleep disorder, the correlation between any two independent variables in the logistic regression equation should be less than 0.8. Skewed distribution data were log-transformed to obtain a normal distribution. Odds ratios with 95% CIs were calculated. All statistical analyses were performed with SPSS version 24.0, and a *p-*value ≤0.05 was considered statistically significant.

## Results

### Incidence of maternal depression, anxiety, and sleep disorder in the third trimester of pregnancy and postpartum

The incidence of maternal depression, anxiety, and sleep disorder in the third trimester of pregnancy was 29.3, 19.7 and 51.3%, respectively. The incidence of maternal postpartum depression, anxiety, and sleep disorder was 28.5, 14 and 67.4%, respectively. The incidence of depression, anxiety, and sleep disorder which extend from the third trimester of pregnancy to postpartum was 13.3, 8.0 and 43.3%, respectively. The incidence of depression, anxiety, and sleep disorder of father was 26.8, 8.9 and 34.5%, respectively. There was a positive correlation between the incidence of maternal depression, anxiety, and sleep disorder in the third trimester of pregnancy and those in the postpartum period. And a correlation can be found in depression, anxiety, and sleep disorder between father and mother.

### Relationship between variables and infant sleep disorder

Infant variables, including the time after birth for filling the BISQ, GA, gender, feeding, rooming-in with mothers, vitamin D intake, and sunlight, did not influence the sleep of infants (Table 2). However, parent and family variables could influence infant sleep disorders. The education level of the father was found to influence the sleep of infants. The higher the father’s education, the less the incidence of sleep disorders in infants. However, other parental and family variables, including age of parents, the BMI, gestational diabetes mellitus, hypertensive disorder complicating pregnancy, and anemia of the mother, family income, residential area, and smoking, did not influence the sleep of infants (Table 3).

**Table 2:** Variables of infants with and without sleep disorders

Variables	N	Without sleep disorder	With sleep disorder	*P*
–	513	305 (59.5%)	208 (40.5%)	–
Time (day)	44 (40–51)	44 (41–51)	43 (40–48)	0.058
GA (week)	39.286 (38.571–40.142)	39.428 (38.571–40.142)	39.285 (38.571–40.142)	0.83
BW (kg)	3.20 (2.95–3.45)	3.20 (2.95–3.45)	3.15 (2.95–3.45)	0.92
Gender				0.187
Male	268 (52.2)	152 (56.7)	116 (43.3)	
Female	245 (47.8)	153 (62.4)	92 (37.6)	
Delivery				0.851
Vaginal	286 (55.8)	169 (59.1)	117 (40.9)	
Cesarean	227 (44.2)	136 (59.9)	91 (40.1)	
Asphyxia				0.367
No	505 (98.4)	299 (59.2)	206 (40.8)	
Yes	8 (1.6)	6 (75)	2 (25)	
Hospitalized				0.205
No	473 (92.2)	285 (60.3)	188 (39.7)	
Yes	40 (7.8)	20 (50)	20 (50)	
Feeding				0.393
Breast milk	297 (57.9)	184 (62)	113 (38)	
Bottle milk	78 (15.2)	43 (55.1)	35 (44.9)	
Both	138 (26.9)	78 (56.5)	60 (43.5)	
Breast milk				0.289
No	437 (85.2)	264 (60.4)	173 (39.6)	
Yes	76 (14.8)	41 (53.9)	35 (46.1)	
Rooming-in				0.957
No	17 (3.3)	10 (58.8)	7 (41.2)	
Yes	496 (96.7)	295 (59.5)	201 (40.5)	
Allergy				0.312
No	413 (80.5)	250 (60.5)	163 (39.5)	
Yes	100 (19.5)	55 (55)	45 (45)	
Jaundice				0.166
No	240 (46.8)	135 (56.3)	105 (43.8)	
Yes	273 (53.2)	170 (62.3)	103 (37.7)	
Phototherapy				0.797
No	434 (84.6)	257 (59.2)	177 (40.8)	
Yes	79 (15.4)	48 (60.8)	31 (39.2)	
Vitamin D				0.985
No	126 (24.6)	75 (59.5)	51 (40.5)	
Yes	387 (75.4)	230 (59.4)	157 (40.6)	
Sunlight				0.767
No	186 (36.3)	109 (58.6)	77 (41.4)	
Yes	327 (63.7)	196 (59.9)	131 (40.1)	

Time: time which the infant’s mother filled out the BISQ. *GA* gestational age, *BW* birth weight, *Hospitalized* infant was in the hospital in the neonatal period, *Vitamin D* vitamin D intake, *Sunlight* infant had sunlight before filling out the BISQ

**Table 3:** Parents and family variables of infants with and without sleep disorders

Variables	N	Without sleep disorder	With sleep disorder	*P*
–	513	305 (59.5%)	208 (40.5%)	
**Mother**				
Age (year)	29 (27–32)	29 (27–32)	29 (27–32)	0.970
Education				0.071
Non-high	357 (69.6)	203 (56.9)	154 (43.1)	
High	156 (30.4)	102 (65.4)	54 (34.6)	
BMI-1	20.3 (18.51–22.41)	20.40 (18.51–22.81)	20.07 (18.51–22.02)	0.25
BMI-2	25.48 (23.44–27.84)	25.65 (23.63–27.91)	25.24 (23.31–27.64)	0.476
BMI-3	22.31 (20.45–24.48)	22.31 (20.68–24.55)	22.27 (20.22–24.41)	0.773
GDM				0.862
No	405 (78.9)	240 (59.3)	165 (40.7)	
Yes	108 (21.1)	65 (60.2)	43 (39.8)	
HDCP				0.280
No	474 (92.4)	285 (60.1)	189 (39.9)	
Yes	39 (7.6)	20 (51.3)	19 (48.7)	
Anemia				0.384
No	378 (73.7)	229 (60.6)	149 (39.4)	
Yes	135 (26.3)	76 (56.3)	59 (43.7)	
**Father**				
Age (year)	30 (28–34)	30 (28–34)	30 (28–35)	0.599
BMI	23.03 (20.76–25.4)	23.11 (20.79–25.36)	22.86 (20.76–25.35)	0.604
Education				0.03
Non-high	365 (71.2)	202 (55.3)	163 (44.7)	
High	148 (28.8)	103 (69.6)	45 (30.4)	
**Family**				
Income				0.485
Low	242 (47.2)	140 (57.9)	102 (42.1)	
High	271 (52.8)	165 (60.9)	106 (39.1)	
Residential Area				0.11
Low	289 (56.3)	163 (56.4)	126 (43.6)	
High	224 (43.7)	142 (63.4)	82 (36.6)	
Smoking				0.12
No	191 (37.2)	122 (63.9)	69 (36.1)	
Yes	322 (62.8)	183 (56.8)	139 (43.2)	

*BMI* body mass index, *BMI-1* BMI before pregnancy, *BMI-2* BMI before delivery, *BMI-3* BMI 1 month after delivery, *GDM* gestational diabetes mellitus, *HDCP* hypertensive disorder complicating pregnancy; Income: less than 5000 yuan is defined as low income, Residential Area: less than 120 m^2^ is defined as a low residential area

### Relationship between parental emotion and sleep disorders and infant sleep disorder

As shown in Table 4, postpartum maternal depression, anxiety, and sleep disorder may be related to the high incidence of infant sleep disturbances, but maternal depression, anxiety, and sleep disorder occurring in the third trimester of pregnancy did not affect infant sleep. Infants whose mothers had sleep disorder extend from the third trimester to postpartum could have higher incidence of sleep disorder than infants whose mothers without sleep disorder. There is a relationship between father’s depression emotion and infants sleep disorder.

**Table 4:** Parental emotion and sleep conditions of infants with and without sleep disorders

Variables	N	Without sleep disorder	With sleep disorder	*P*
–	513	305 (59.5%)	208 (40.5%)	
**Third trimester**				
Time (week)		35 (34–37)	36 (34–37)	0.166
SDS Score	46.32 ± 9.01	45 (39–55)	47 (42–53)	0.306
Depression				0.686
No	362 (70.6)	218 (60.2)	144 (39.8)	
Yes	151 (29.4)	87 (57.6)	64 (43.2)	
SAS Score	41 (35–47)	40 (33–46)	45 (36–48)	0.041
Anxiety				0.491
No	417 (81.3)	253 (60.7)	164 (39.3)	
Yes	96 (18.7)	52 (54.2)	44 (45.8)	
PSQI Score	7 (4.75–10)	6 (5–10)	7 (4–10)	0.747
Sleep disorder				0.119
No	249 (48.5)	164 (65.8)	85 (34.2)	
Yes	264 (51.5)	141 (53.4)	123 (46.6)	
**After delivery**				
Time (day)	44 (40–51)	44 (41–51)	43 (40–48)	0.058
SDS Score	45 (36–55)	43 (35.5–52)	50 (38–60)	< 0.001
Depression				< 0.001
No	367 (71.5)	238 (64.9)	129 (35.1)	
Yes	146 (28.5)	67 (45.9)	79 (54.1)	
SAS Score	37 (31–44)	36 (30–42)	40 (33–47)	< 0.001
Anxiety				0.050
No	441 (86)	273 (61.9)	168 (38.1)	
Yes	72 (14)	32 (44.4)	40 (55.6)	
PISQ Score	8 (6–11)	7 (5–10)	9.5 (7–12)	< 0.001
Sleep disorder				< 0.001
No	167 (32.6)	121 (72.5)	46 (27.5)	
Yes	346 (67.4)	184 (53.2)	162 (46.8)	
**Both periods**				
Depression				0.361
No	445 (86.7)	270 (60.8)	175 (39.2)	
Yes	68 (13.3)	35 (51.5)	33 (50.0)	
Anxiety				0.406
No	509 (99.2)	301 (59.1)	208 (40.9)	
Yes	4 (0.8)	4 (100)	0	
Sleep disorder				0.011
No	291 (56.7)	198 (68.0)	93 (32.0)	
Yes	222 (43.3)	107 (48.2)	115 (51.8)	
**Father**				
Time (day)	44 (40–51)	44 (41–51)	43 (40–48)	0.058
SDS Score	44.11 ± 11.9	42.62 ± 11.95	46.36 ± 11.55	0.045
Depression				0.277
No	376 (73.3)	232 (61.7)	144 (38.3)	
Yes	137 (26.7)	73 (53.3)	64 (46.7)	
SAS Score	36.91 ± 9.08	36.5 ± 9.02	37.52 ± 9.22	0.481
Anxiety				0.587
No	467 (91.0)	275 (58.9)	192 (41.1)	
Yes	46 (9.0)	30 (65.2)	16 (34.8)	
PISQ Score	5.43 ± 3.46	5.11 ± 3.27	5.93 ± 3.71	0.146
Sleep disorder				0.342
No	336 (65.5)	208 (61.9)	128 (38.1)	
Yes	177 (34.5)	97 (54.8)	80 (45.2)	

Time: time of measurement, Both periods: mothers had emotion or sleep disorders extend from the third trimester of pregnancy to postpartum

### Risk factors for infant sleep disorder

According to the relationships between variables and infant sleep disorder in Tables 2, 3, and 4, we selected the time for mother to fill out the BISQ, education of father, education of mother, depression of father, maternal postpartum depression, postpartum anxiety, postpartum sleep disorder, and maternal sleep disorder which extend from the third trimester of pregnancy to postpartum for logistic regression analysis of infant sleep disorder. Because the correlations among depression of father, postpartum depression of mother, and postpartum anxiety of mother were higher than 0.8, they should be performed in the logistic regression analysis for risk factors of infant sleep disorder, respectively. The results (Table 5) showed that the risk factors of infant sleep disorder were low education level of the father (Model 1: RR = 0.537, 95%CI = 0.323–0.895; Model 2: RR = 0.542, 95%CI = 0.326–0.9), depression of father (Model 3: RR = 1.03, 95%CI = 1.001–1.06), postpartum depression of mother (Model 1: RR = 1.891, 95%CI = 1.261–2.837), postpartum anxiety of mother (Model 2: RR = 1.699, 95%CI = 1.009–2.86), postpartum sleep disorder of mother (Model 1: RR = 2.193, 95%CI = 1.452–3.312; Model 2: RR = 2.239, 95%CI = 1.483–3.38), and maternal sleep disorder extend from the third trimester of pregnancy to postpartum (Model 1: RR = 2.525, 95%CI = 1.272–5.012; Model 2: RR = 2.632, 95%CI = 1.286–5.143).

**Table 5:** Logistic regression of the risk factors for infant sleep disorder

	Model 1			Model 2			Model 3		
Variables	*OR*	95% *CI*	*P*	*OR*	95% *CI*	*P*	*OR*	95% *CI*	*P*
Time	0.987	0.971–1.003	0.102	0.987	0.971–1.002	0.094	0.983	0.956–1.01	0.217
Education of father	0.537	0.323–0.895	0.017	0.542	0.326–0.9	0.018	0.474	0.184–1.216	0.12
Education of mother	1.029	0.624–1.696	0.911	0.952	0.581–1.56	0.846	1.513	0.583–3.925	0.395
Postpartum depression	1.891	1.261–2.837	0.002	–	–	–	–	–	–
Postpartum anxiety	–	–	–	1.699	1.009–2.86	0.046	–	–	–
Postpartum sleep disorder	2.193	1.452–3.312	< 0.001	2.239	1.483–3.38	< 0.001	1.194	1.079–1.322	0.001
Both periods sleep disorder	2.525	1.272–5.012	0.008	2.632	1.286–5.143	0.004	–	–	–
Depression of father	–	–	–	–	–	–	1.03	1.001–1.06	0.042

Time: the time when the infant’s mother filled out the BISQ, Both periods sleep disorder: mothers had sleep disorders extend from the third trimester of pregnancy to postpartum

The incidence of infant sleep disorder was 30.4% in those infants whose fathers had high education levels (University education level or higher), but it was increased to 44.7% in those infants whose father had low education levels. For infants whose mothers had postpartum depression, postpartum anxiety, or postpartum sleep disorder, the incidence of infant sleep disorder rose from 35.1, 38.1%, and 27.5 to 54.1, 55.6, and 46.8%, respectively. Moreover, for the mother who had sleep disorder extend from the third trimester of pregnancy to postpartum, the incidence of infant sleep disorder was 51.8%.

Babies whose mothers were depressed after delivery had about 1.9 times the risk of developing sleep disorder compared with those whose mothers were not depressed. Babies whose mothers had postpartum anxiety had about 1.7 times the risk of sleep disorder compared with those whose mothers had no postpartum anxiety. Babies whose mothers had postpartum sleep disorders had a 2.2-fold higher risk of developing sleep disorders than those whose mothers had no postpartum sleep disorders. The risk of sleep disorders in infants whose mothers had sleep disorders extend from the third trimester to postpartum was about 2.5 ~ 2.6 times higher than that in infants whose mothers had no sleep disorder.

### Expression of DR, GR, MR, and EPAC in the placenta

**Fig. 1:**
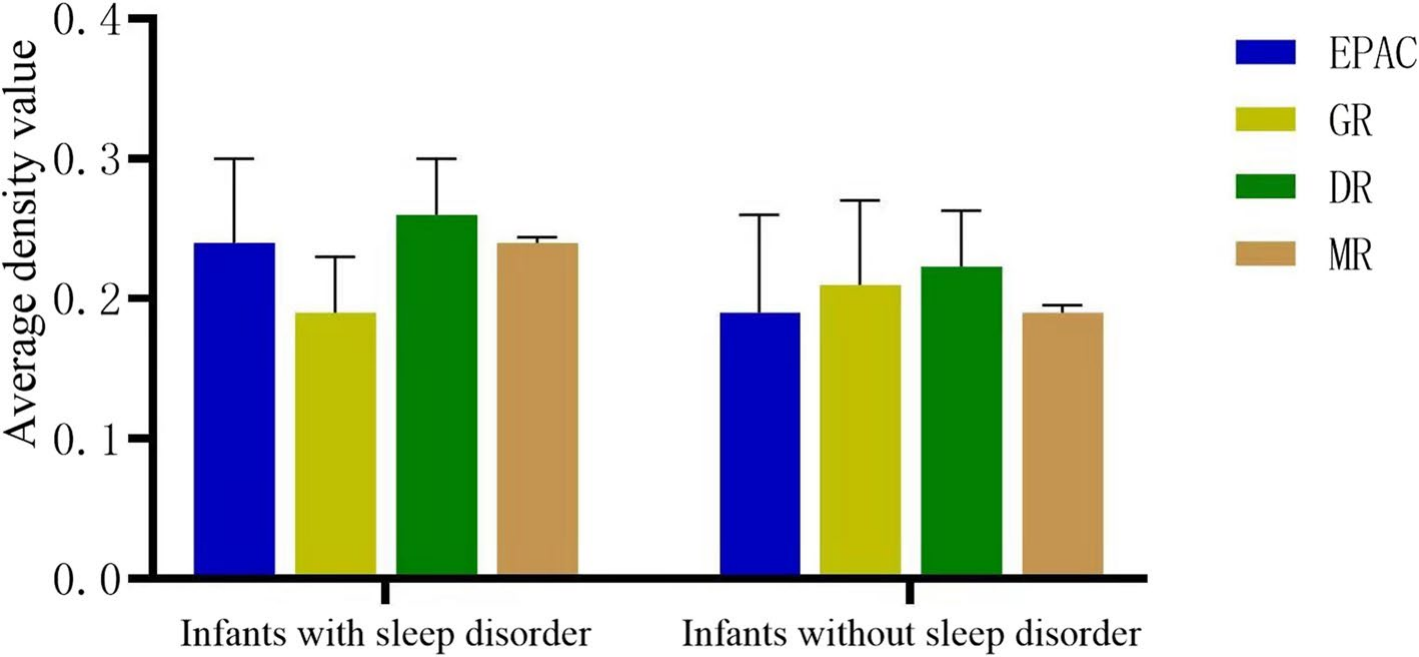
Comparison of DR, GR, MR, and EPAC expression in the placentas of mothers with and without infants sleep disorders. EPAC: exchange proteins directly activated by cAMP receptor; GR: glucocorticoid receptor; DR: dopamine receptor; MR: melatonin receptor

**Fig. 2:**
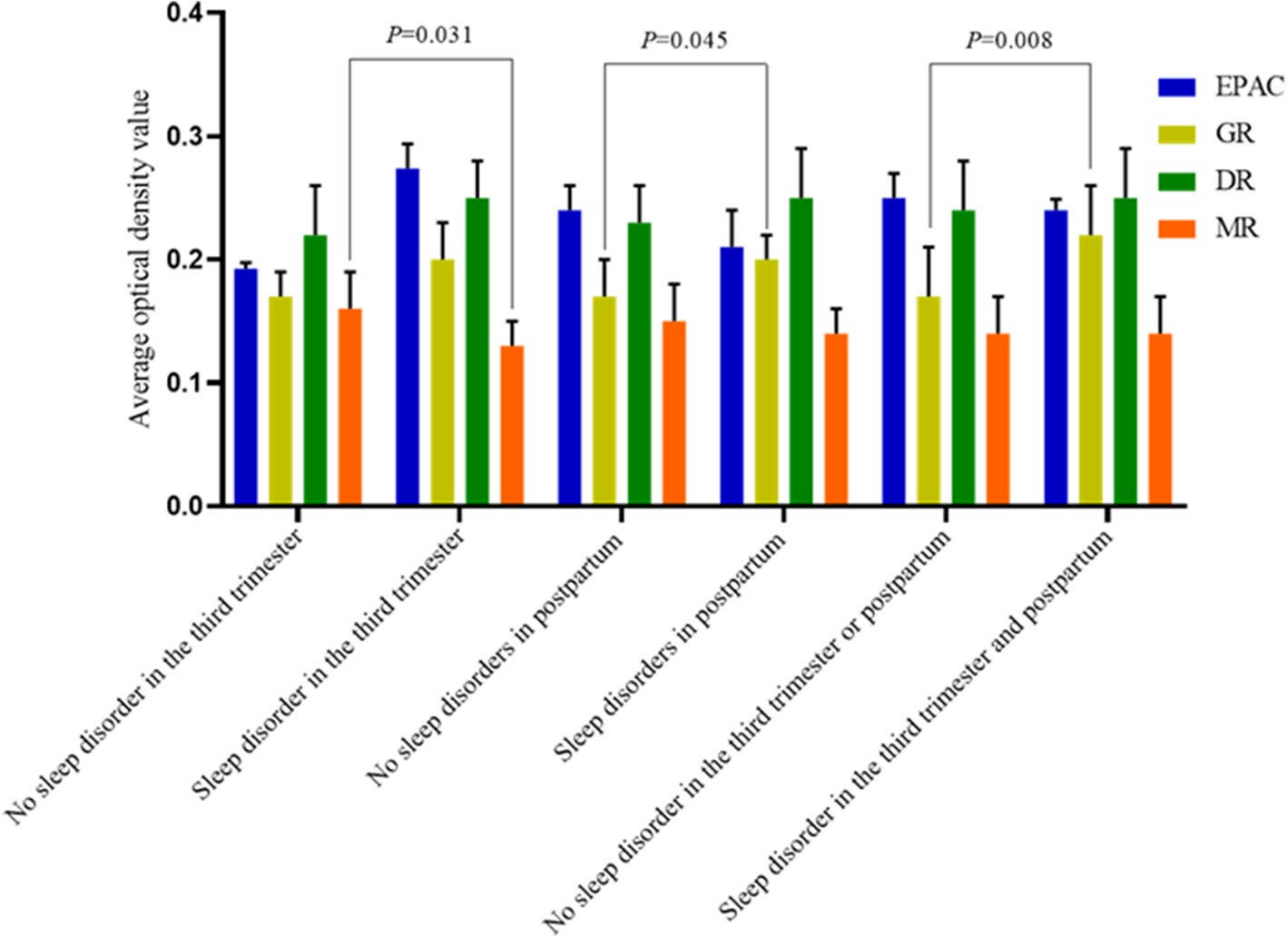
Comparison of DR, GR, MR, and EPAC expression in the placentas of mothers with different sleep conditions. EPAC: exchange proteins directly activated by cAMP receptor; GR: glucocorticoid receptor; DR: dopamine receptor; MR: melatonin receptor

Expression of DR, GR, MR, and EPAC in the placenta are shown in Figs. 1 and 2. There was no difference in expression of DR, GR, MR, and EPAC in the placentas of mothers whose infants had sleep disorders compared with those placentas from mothers whose infants without sleep disorders (see Fig. 1). The content of MR in the placenta of pregnant women with sleep disorder in the third trimester was lower than that of the group without sleep disorder (see Fig. 2), which indicated that sleep disorder in the third trimester could reduce the expression of MR in the placenta. The level of GR expression in the placenta of mother with postpartum sleep disorders was higher than that of the group without sleep disorders (see Fig. 2). Higher expression of GR in the placenta of mother with sleep disorder extend from the third trimester of pregnancy to postpartum, suggesting that increased placental GR expression caused by sleep disorder in the third trimester of pregnancy, could promote the occurrence of postpartum sleep disorders.

### Methylation of the promoter regions of the placental DR, GR, MR, and EPAC genes in mothers with and without sleep disorder

Due to the expense of NGS-BSP, we used a nested case-control study to select specimens. Expression of MR and GR were different between mothers with and without sleep disorder, so placentas were divided into four groups according to the mother’s sleep characteristics: (1) sleep disorder extend from the third trimester of pregnancy to postpartum (Group 1), (2) sleep disorder in the third trimester only (Group 2), (3) postpartum sleep disorder (Group 3), and (4) no sleep disorder (Group 4). There were 36 samples in each group. We compared some factors that may affect the expression of MR and GR, including fetal sex, gestational age, birth weight, delivery method, HDCP, pregnancy diabetes, BMI of mother, education level, income, SAS score, and SDS score. There was no difference in the above-mentioned factors among the above four groups.

**Fig. 3:**
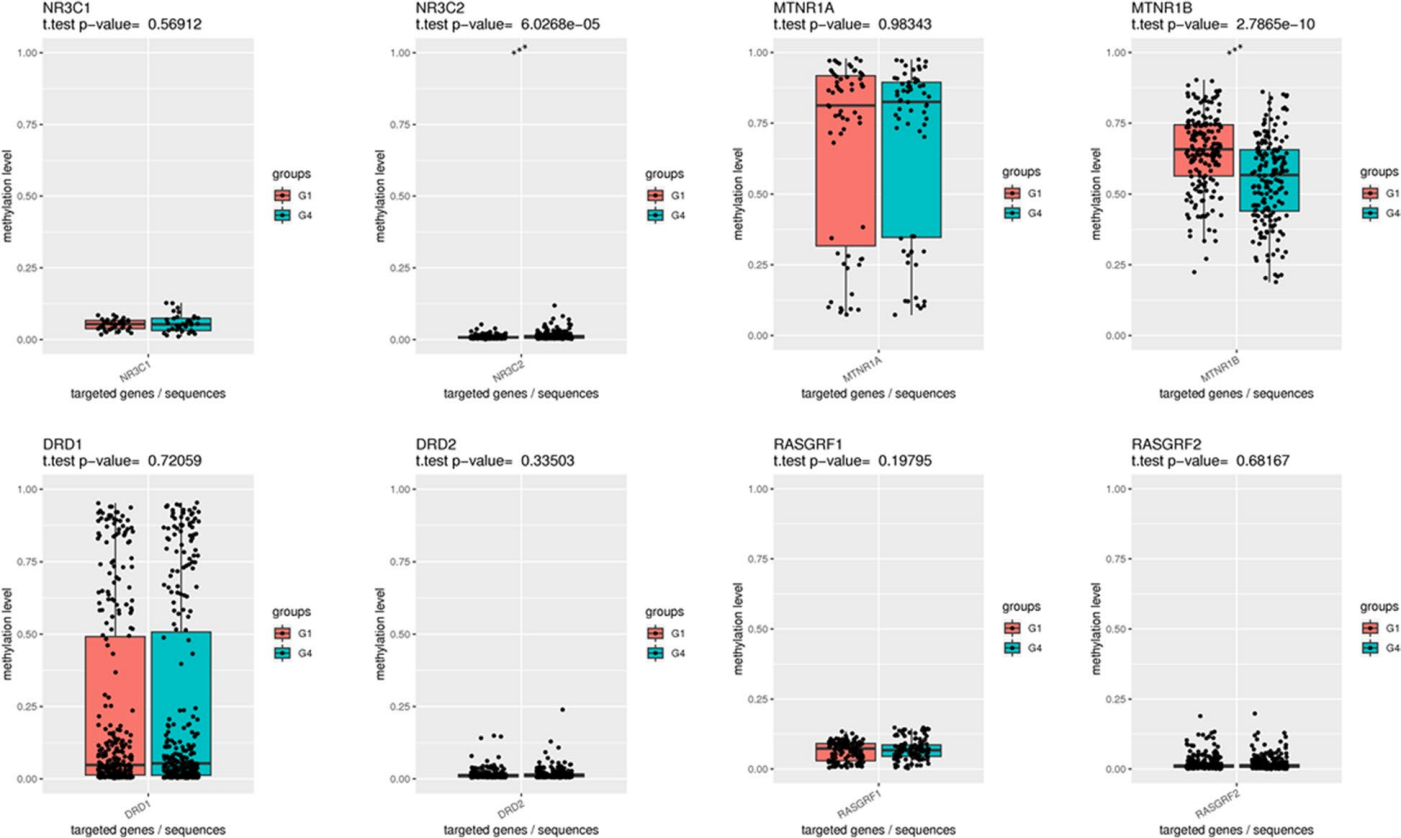
Comparison of DNA methylation levels of placental DR, GR, MR, and EPAC genes between mothers in Group 1 and Group 4. Group 1: sleep disorder which extend from the third trimester of pregnancy to postpartum; Group 4: No sleep disorder. The paired t-test was used for statistical analysis (*P < 0.05, **P < 0.01, ***P < 0.001). EPAC: exchange proteins directly activated by cAMP receptor; GR: glucocorticoid receptor; DR: dopamine receptor; MR: melatonin receptor. NR3C1 and NR3C2 are the genes of GR; MTNR1A and MTNR1B are the genes of MR; DRD1 and DRD2 are the genes of DR; RAGRF 1 and RAGRF 2 are the genes of EPAC

**Fig. 4:**
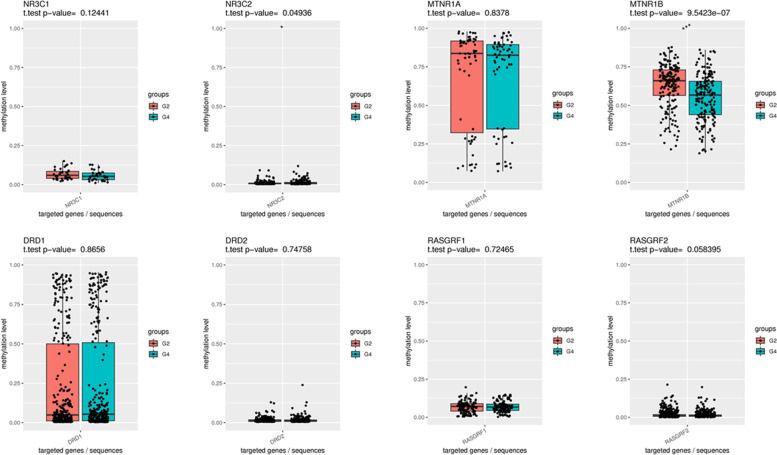
Comparison of DNA methylation levels of placental DR, GR, MR, and EPAC genes between pregnant women in Group 2 and Group 4. Group 2: Sleep disorder in third trimester only; Group 4: No sleep disorder. The paired t-test was used for statistical analysis (*P < 0.05, **P < 0.01, ***P < 0.001). EPAC: exchange proteins directly activated by cAMP receptor; GR: glucocorticoid receptor; DR: dopamine receptor; MR: melatonin receptor. NR3C1 and NR3C2 are the genes of GR; MTNR1A and MTNR1B are the genes of MR; DRD1 and DRD2 are the genes of DR; RAGRF 1 and RAGRF 2 are the genes of EPAC

**Fig. 5:**
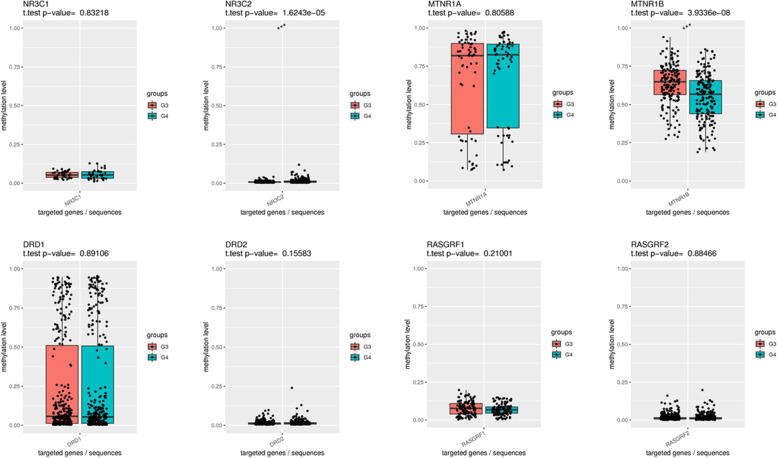
Comparison of DNA methylation levels of placental DR, GR, MR, and EPAC genes between pregnant women in Group 3 and Group 4. Group 3: Postpartum sleep disorder; Group 4: No sleep disorder. The paired t-test was used for statistical analysis (*P < 0.05, **P < 0.01, ***P < 0.001). EPAC: exchange proteins directly activated by cAMP receptor; GR: glucocorticoid receptor; DR: dopamine receptor; MR: melatonin receptor. NR3C1 and NR3C2 are the genes of GR; MTNR1A and MTNR1B are the genes of MR; DRD1 and DRD2 are the genes of DR; RAGRF 1 and RAGRF 2 are the genes of EPAC

Methylation of the promoter regions of the placental DR (DRD1 and DRD2), GR (NR3C1 and NR3C2), MR (MTNR1A and MTNR1B), and EPAC (RASGRF1 and RASGRF2) genes are shown in Figs. 3, 4, 5. Methylation of the promoter regions of the placental NR3C2 were lower in the mothers with sleep disorder (Group 1, Group 2, and Group 3) than that of the group without sleep disorder (Group 4). Methylation of MTNR1B were higher in the placenta of mothers with sleep disorder (Group 1, Group 2, and Group 3) than that of the group without sleep disorder (Group 4). There was no difference in the methylation of the promoter regions of the placental DR (DRD1 and DRD2), GR (NR3C1), MR (MTNR1A), and EPAC (RASGRF1 and RASGRF2) genes in mothers with sleep disorders compared with those without sleep disorders.

## Discussion

In this study cohort, the incidences of depression, anxiety, and sleep disorder in mothers are close to other studies [19, 20]. In addition, we found a positive correlation between depression, anxiety, and sleep disorder in the third trimester of pregnancy and postpartum. The incidence of sleep disorders in infants 0–3 months of age in this cohort was 40.5%, which is similar to the incidence of infant sleep problems in other cities of China [21, 22]. Thus, our study population in this cohort has similar representation as in previous studies.

In this study, we found high education level of father is the protection factor of infant sleep, which is in line with another study in China [23]. The higher education level of father may improve the baby rearing patterns, thus protecting the baby sleep quality, but the mechanism needs further exploration in future study. We also found postpartum depression, anxiety, and sleep disorders of mothers are related to infant’s sleep pattern, and there is a relationship between father’s depression emotion and infants sleep disorder. Studies have shown that mothers with postpartum depression have poor ability to understand and respond to infant signals, and show more negative effects on infants, and are more intrusive in their interactions with infants [24, 25]. In addition, mothers with sleep disorders have a higher degree of worry about their infants’ physical and emotional health [26], and such excessive worry may increase the intervention on infants at night, leading to more waking times of infants and then increasing the risk of infant sleep disorders. On the other hand, when infants have sleep disorders, their mothers’ sleep at night is also interrupted, and this effect is more obvious when mothers have depression, indicating that infant sleep disorders will also increase the risk of postpartum emotional and sleep disorders in mothers [26, 27]. Something is similar in the relationship between father’s depression emotion and infants sleep disorder. There is a study suggested that sleep disorders in infants could increase the risk of depression in fathers [28].

Multivariate analysis showed that maternal sleep disorder in the third trimester of pregnancy had no effect on infant sleep in this cohort study, which is different from other studies [5]. In this study, we found placental MR expression of mothers with sleep disorder in the third trimester is lower than that without sleep disorder, but there was no difference in the expression of DR, GR, MR, and EPAC in the placenta of mothers whose infants have and do not have sleep disorders. We believe that maternal sleep disorder in third trimester does not directly increase the risk of infant sleep disorder, but it is not clear whether changes in placental MR expression could increase the risk of postpartum sleep disorders in mothers. Therefore, we performed the correlation analysis maternal sleep disorders in the third trimester of pregnancy and postpartum, which showed that sleep disorders in the third trimester of pregnancy can increase the risk of postpartum sleep disorders in mothers. Furthermore, we found maternal sleep disorder in the third trimester of pregnancy results in decreased NR3C2 promoter methylation and increased MNTR1B promoter methylation. Studies have shown that hyperactivity of the HPA axis increases the risk of sleep disorders [29]. Melatonin has an inhibitory effect on activity of the HPA axis [7]. When the methylation of MTNR1B in the placenta increases in the late trimester of pregnancy, MR expression decreases, resulting in decreased inhibition by melatonin on the maternal HPA axis, which increases cortisol secretion. Increased cortisol levels will activate GR receptors to over-activate the HPA axis. The methylation of NR3C2 and MNTR1B were similarly altered in the placenta of mother with postpartum sleep disorders. Therefore, these findings suggest that maternal sleep disorders in late pregnancy of pregnancy could increase the risk of postpartum sleep disorders by changing placental NR3C2 and MNTR1B methylation levels to result in decreased expression of MR and increased expression of GR in the placenta.

The results of this study show that the risk of sleep disorder in infants whose mothers with sleep disorder extend from the third trimester of pregnancy to postpartum is higher than those whose mothers without sleep disorder. Moreover, sleep disorders occurring extend from the late trimester of pregnancy to postpartum have a greater impact on infant sleep than sleep disorders occurring in the late trimester or postpartum alone, suggesting the cumulative effect of maternal sleep disorders on infant sleep. We examined the placenta of mothers with sleep disorder, occurring extend from the third trimester of pregnancy to postpartum, and showed that NR3C2 methylation decreased and MTNR1B methylation increased, which supports the hypothesis that maternal sleep disorders in the third trimester of pregnancy could increase maternal postpartum sleep disorder by changing placental NR3C2 and MNTR1B methylation levels. Postpartum sleep disorders in mothers could increase the risk of infant sleep disorders [24, 25]. The above results suggest that sleep disorders of mothers in the third trimester of pregnancy can lead to decreased expression of MR, resulting in elevated cortisol levels to activate an increased number of GRs that result from down-regulation of NR3C2 promoter methylation to increase the risk of postpartum sleep disorders [30, 31], and then maternal postpartum sleep disorder could increase infants sleep disorder in turn.

However, this study was a single-center cohort study with small sample size, which may lead to selection bias. In addition, the results which we showed above may be one of the mechanisms that maternal emotion and sleep conditions affect infant sleep. We should expand the sample size in the future study, and then further explore the mechanism of parental emotion and sleep conditions affecting infant sleep.

## Conclusion

This study found that postpartum sleep disorders of mothers could directly increase the risk of infant sleep disorders. In addition, maternal sleep disorders occurring extend from the third trimester of pregnancy to the postnatal period impact infant sleep seriously. Infant sleep disorders can also aggravate postpartum sleep disorders. Therefore, we should pay attention to maternal emotion and sleep conditions during pregnancy and postpartum and focus on infant sleep conditions when preforming child health care. Early detection and intervention of maternal sleep disorders in the late pregnancy can reduce the postpartum sleep disorders of the mothers, to subsequently decrease the incidence of infant sleep disorder.

## Data Availability

The data in this study are available from the corresponding author on reasonable request.
